# On the relationship between emotional state and abnormal unfairness sensitivity in alcohol dependence

**DOI:** 10.3389/fpsyg.2015.00983

**Published:** 2015-07-09

**Authors:** Damien Brevers, Xavier Noël, Catherine Hanak, Paul Verbanck, Charles Kornreich

**Affiliations:** ^1^Department of Psychology, Brain and Creativity Institute, University of Southern California, Los Angeles, CA, USA; ^2^Psychological Medicine Laboratory, Faculty of Medicine, Université Libre de Bruxelles, Brussels, Belgium

**Keywords:** alcohol dependence, social decision-making, unfairness, electrodermal response

## Abstract

Recent empirical findings suggest that alcohol dependence is characterized by heightened sensitivity to unfairness during social transactions. The present study went a step further and aimed to ascertain whether this abnormal level of sensitivity to unfairness is underlined by an increased emotional reactivity. Twenty-six recently abstinent alcohol-dependent (AD) individuals and 32 controls performed an ultimatum game (UG), in which participants had to respond to take-it-or-leave-it offers, ranging from fair to unfair and made by a fictive proposer. Emotional state was recorded during UG offers presentation and was indexed by the amplitude of skin conductance response (SCR). Results showed that AD decided to reject unfair offers more frequently than their controls, confirming previous data. The proportion of rejected unfair UG offers was correlated with SCR, in the AD but not in the control group. This finding suggests that deciding to accept or reject unfair UG offers is influenced by arousal-affective activity in AD, but not in controls. Heightened emotional reactivity may have driven AD to punish the proposer rather than acting as a rational economic agent. An implication of present findings is that AD might have difficult to cope with unfair situations triggered by social interactions. Future studies are needed in order to examine whether—emotional and behavioral—reactivity to unfairness during the UG could impact alcohol consumption and relapse in AD.

## Introduction

Alcohol-dependent (AD) individuals persevere in alcohol use despite encountering long-term aversive consequences directly linked to their drinking (Noël et al., 2010; American Psychiatric Association, 2013; Camchong et al., 2014). The persistence of such maladaptive habits might be underlined by poor decision-making ability. Indeed, abnormal profiles of decision-making have been repeatedly evidenced in laboratory settings, wherein recently detoxified AD exhibited preference toward short-term uncertain reward over safer long-term reward (Noël et al., 2007; Kornreich et al., 2013; Brevers et al., 2014). Hence, one could infer that the occurrence of such impaired decision-making processes might hamper ADs’ ability to exert willpower, that is, to decide based on both short-term and long-term consequences of an action (Bechara, 2005; Noël et al., 2010).

One limitation of these studies is that they focus on situations where decision outcomes only affect the deciding agent. Hence, those findings cannot be generalized to situations that involve social interactions, that is, social decision-making that affects others as well as the individual who makes the choice (Fehr and Camerer, 2007; Rilling and Sanfey, 2011). Abnormal social decision-making has been evidenced in alcohol dependence through the use of “moral dilemmas,” which trigger a conflict between what is good for the majority (utilitarian judgment) and emotional factors (Greene et al., 2001). Compared to healthy controls, AD would be more prone to choose the rational options (i.e., utilitarian moral judgments) when faced with emotionally salient moral personal dilemmas (Carmona-Perera et al., 2012, 2013, 2014; Khemiri et al., 2012; but see Kornreich et al., 2013). Moreover, by contrast to control participants, AD showed a blunted emotional reactivity (estimated with heart rate response) to moral personal dilemmas (Carmona-Perera et al., 2013). These findings suggest that AD failed to engage emotional aversive reactions within situations of social interactions that involve personal moral violations for others.

Impaired social-decision making has also recently been evidenced in AD through a measure of sensitivity to unfairness. Indeed, a recent study (Brevers et al., 2013) has shown that, as compared to healthy controls, ADs were more prone to reject unfair offers during the ultimatum game (UG; Güth et al., 1982; Sanfey et al., 2003). The context is very different from the studies involving moral dilemmas, as in the UG paradigm, the subjects are confronted with unfair situations directed toward themselves, rather than involving other persons described in theoretical stories. In the UG, two players (a proposer and a responder) have to divide a sum of money in a single trial. If the responder accepts the offer made by the proposer, the deal is validated. On the other hand, if the responder rejects the offer, neither player gets anything. According to rational economic agent, the only way for the responder to maximize profits during the UG is to always accept the offer (Haselhuhn and Mellers, 2005; Hewig et al., 2011). Nevertheless, researchers have constantly observed that individuals are unwilling to accept inequitable UG offers, as compared to fair or medium-fair offers (Camerer and Thaler, 1995; Haselhuhn and Mellers, 2005; Knafo et al., 2008).

Findings from recent studies suggest that the rejection of unfair UG offers is modulated by (negative) emotional processes. More specifically, possible explanation for these seemingly “irrational” rejection is that negative emotions, such as unfairness, might drive participants to punish the proposer rather than making an utilitarian choice (Pillutla and Murnighan, 1996; Fehr and Gächter, 2002). This assumption is in line with the theory of *altruistic punishment* (Trivers, 1971; Fehr and Gächter, 2000, 2002), which advances that people are willing to punish non-cooperators in order to enhance reciprocity in cooperation transactions, even at personal costs to the punisher (as in the case of rejecting an UG offer). Consistent with this theoretical view, inequitable proposals were found to elicit a stronger neural activation in emotion-related brain regions (Sanfey et al., 2003; Koenigs and Tranel, 2007; Feng et al., 2014) and a greater-magnitude of skin conductance response (SCR; van’t Wout et al., 2006; Hewig et al., 2011; but see Osumi and Ohira, 2009). Moreover, the proportion of rejected unfair UG offers is positively associated with self-reported anger (Pillutla and Murnighan, 1996) and with greater SCR (Civai et al., 2010; Wu et al., 2013).

The present study aimed to examine the association between emotional state and unfairness sensitivity in AD and healthy controls while performing the UG. Emotional state was indexed by skin conductance reactivity amplitude. Electrodermal response is a valid measure of emotional activation (Bouscein, 1992; Canli and Lesch, 2007), which has also been found to be sensitive to the degree of unfairness associated with UG offers (van’t Wout et al., 2006; Civai et al., 2010; Hewig et al., 2011; Wu et al., 2013). We hypothesized that (i) AD reject unfair offers more frequently than controls; (ii) in both AD and controls, facing unfair offers will trigger higher SCR, as compared with medium-fair and fair offers; (iii) AD exhibit higher SCR than controls when facing unfair offers.

## Materials and Methods

### Participants and Recruitment

All subjects were adults. Demographics for the two groups are presented in Table 1. All participants provided informed consent. The study was fully approved by the Ethics Committee of the Brugmann University Hospital and conducted in accordance with the Declaration of Helsinki. For the AD group, medical histories were obtained by interview by a board-certified psychiatrist.

**TABLE 1:** **Demographic data means and standard deviations for the alcohol-dependents (ADs) and controls**.

	**AD**	**Control**
*N*	26	32
Age (years)	42.53 (8.83)	41.25 (10.08)
Gender (male/female)	19/7	24/8
Education (years)	13.42 (3.17)	17.06 (3.14)****
Duration of alcohol abuse (years)	13.50 (10.99)	–
Mean alcohol use (drinks/day)	15.03 (10.81)	1.96 (1.82)****
Number of prior hospitalizations for alcohol detoxification	2.61 (2.51)	–
Tobacco use (number of cigarettes per day)	24.15 (16.81)	3.43 (5.76)****
AUDIT	28.89 (5.20)	2.59 (3.87)****
BDI	10.96 (6.23)	3.56 (3.44)****
STAI-S	54.27 (12.25)	32.46 (14.02)****
STAI-T	42.61 (14.02)	30.03 (9.65)****

Values shown are the mean and standard deviation on each measure. AUDIT, Alcohol Use Disorders Identification Test; BDI, Beck Depression Inventory; STAI-S, State subscale of the State-Trait Anxiety Inventory; STAI-T, Trait subscale of the State-Trait Anxiety Inventory. ****t-test p < 0.0001.

Twenty-six AD participants were recruited for this study from the Alcohol Detoxification Unit of the Brugmann University Hospital (Brussels). They were tested in their third week of alcohol detoxification (i.e., the week before their detoxification program end). They all received complete medical, neurological and psychiatric examinations at the time of the selection. AD participants were all diagnosed (made by PV and CH, board-certified CHU-Brugmann psychiatrists) with alcohol dependence according to DSM-V (American Psychiatric Association, 2013) criteria and confirmed by the Alcohol Use Disorders Identification Test (AUDIT; Saunders et al., 1993). We excluded any subject who reported a lack of comprehension of French language, or who had evidence of schizophrenia and other psychotic disorders, bipolar disorders, polysubstance-related disorders, pathological gambling and overt cognitive dysfunction (based on DSM-V interviews, clinical observations, and anamnestic information collected by PV and CH). Reasons for exclusion were other current DSM–IV Axis I diagnoses, a history of significant medical illness, head injury resulting in a loss of consciousness for longer than 30 min that might have affected the central nervous system, use of other psychotropic drugs or substances that influence cognition and overt cognitive dysfunction. Subjects were examined after they had abstained from alcohol for a minimum of 18 days and at least 5 days after a standard detoxification period. The detoxification regimen consisted of B vitamins and decreasing doses of sedative medication (diazepam). All received complete medical, neurological, and psychiatric evaluations prior to enrolment in the study.

Thirty-two control participants, similar for sex and age, were recruited by word of mouth from healthy community members; they were not paid for their participation. Exclusion criteria were a present Axis I psychiatric diagnosis; substance-use disorder during the year before enrollment in the study; or consumption of more than four standard alcoholic drinks per day for longer than 1 month.

For each participant, current clinical status of depression and anxiety levels were rated with the Beck Depression Inventory (Beck et al., 1961) and the Spielberger State-Trait Anxiety Inventory (STAI-S, STAI-T; Spielberger, 1983). Participants completed the STAI-S just prior performing the UG task.

### Task

**FIGURE 1:**
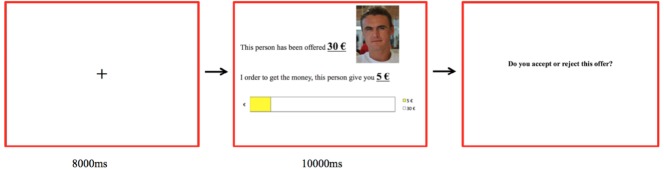
**Timeline for a single trial of the ultimatum game.** The original screens were in French.

Participants played as responders in a series of 54 single round trials of the UG via computer interface. Before the game started, participants were given detailed verbal explanations and confirmed verbally that they understood the game. No real monetary amount was awarded to the participants. At the beginning of each trial, a white-colored, cross-shaped fixation point was presented for 8000 ms in order to allow autonomic activity from the previous trial to recover to baseline. Next, they saw the amount of the stake and the amount proposed by the partner for 10000 ms (see Figure 1). During this time, a picture of the proposer’s face was displayed in the upper right of the computer screen (see Figure 1). Then, a “response cue” was displayed and participants indicated whether they accepted or reject the offer by pressing one of two buttons (labeled “accept” or “reject”) on a button box.

Photographs of 54 faces (27 male, 27 female, Caucasian, with a neutral expression; taken from Brevers et al., 2013) were randomly matched with the offers. There were 18 fair offers (proposition of 40, 45, or 50% of the stake to the responder), 18 medium-fair offers (proposition of 27, 30, or 33% of the stake to the responder) and 18 unfair offers (proposition of 17, 20, or 22% of the stake to the responder). During each session, the order of the offers was randomized.

### Apparatus

The UG paradigm was programmed using E-prime 2.0 Professional (Psychology Software Tools, Sharpsburg, PA, USA). Electrodermal activity (EDA) was recorded during the task using a BIOPAC MP150 (Biopac Systems, Santa Barbara, CA, USA). The BIOPAC was connected to a stimulus delivery computer and a second administrator computer running AcqKnowledge v3.9.0. Events occurring on the stimulus delivery computer were synchronized to the psychophysiology recording using digital channels. EDA was measured using two grounded Ag–AgCl electrodes (a BIOPAC TSD203 transducer with a GSR100C amplifier module; gain = 5 V, low pass filter 1.0 Hz, high pass filters DC), secured on the distal phalange of the index and middle fingers of the non-dominant hand. Isotonic paste (BIOPAC Gel 101, with recommended NaCl concentration of 0.05 M) was used as the electrolyte. The EDA signal was transformed into micro-Siemens units (mS) in Acqknowledge.

### Data Preprocessing

Physiological data were analyzed offline in AcqKnowledge (Biopac Systems, Santa Barbara, CA, USA). SCR amplitude was defined as the change from the baseline to the peak of the response started within a 0.5–4 s duration following offer onset. Behavioral (mean proportion of acceptance) and physiological (mean SCR) was not normally distributed, at each level of type of offers (fair, medium-fair, unfair) and for each decision type (accept vs. reject). Attempts of log transformation (log [SCR + 1]) had no significant effect on the normality of behavioral and physiological data. Therefore, non-parametric statistical tests were performed to analyze behavioral decision and SCR amplitude during the UG.

## Results

### Current Clinical Status

Independent samples *t*-tests revealed that AD participants had lower scores of education, higher scores of depression, trait anxiety and state anxiety (prior to testing). These results are shown in Table 1. However, we found no correlation (Spearman rank) between UG (behavioral and electrodermal) data and the scores of education, depression, trait and state anxiety, for the whole sample (*N* = 58), within the control (*n* = 32) or the alcohol dependence group (*n* = 26). We observed no significant effect of gender (for the whole sample and within the AD and control groups separately) on UG (behavioral and electrodermal) data. There was also no relationship between UG data in the AD group and duration of consumption or mean consumption per day.

### Behavioral Performances

A Friedman test was performed separately in each group in order to examine the effect of the type of offers (fair vs. medium-fair vs. unfair) on the proportion of acceptance (see Table 2, for descriptive statistics). These analyses showed that there were significant differences, in each group, between the fair and the medium-fair offers (*p* < 0.0001), between the fair and the unfair offers (*p* < 0.0001). In the control group, we found no significant difference between the medium-fair and the unfair offers, χ^2^(1,32) = 0.69, *p* = 0.40. By contrast, in the alcohol dependence group, there was a significant difference between the medium-fair and the unfair offers [χ^2^(1,26) = 4.00, *p* = 0.046], indicating that AD participants reject more unfair offers than medium fair offers.

**TABLE 2:** **Descriptive statistics of the proportion of offers accepted for alcohol-dependent and control participants with unfair, medium fair, and fair offers**.

		**Percentiles**		
		**25th**	**Median**	**75th**
Controls	Fair	0.77	100	100
	Medium-fair	0.12	0.79	100
	Unfair	0.08	0.50	100
Alcohol-dependent	Fair	0.48	0.83	100
	Medium-fair	0.17	0.33	0.58
	Unfair	0.00	0.17	0.50

Mann–Whitney U tests were then performed to examine between-groups differences on the proportion of acceptance according to the type of offers. There was a significant difference between AD (Mean Rank = 24.35) and controls (Mean Rank = 33.69) for the unfair offers (Mann–Whitney U statistic = 282.00, *Z* = –2.132, *p* = 0.033), indicating that AD decided to reject unfair offers more frequently than controls (see Table 2, for descriptive statistics). There was no significant difference between AD and control groups for the medium-fair (Mann–Whitney U statistic = 306.50, *Z* = –1.741, *p* = 0.082) and the fair offers (Mann–Whitney U statistic = 323.00, *Z* = –1.546, *p* = 0.122). In addition, in order to estimate the interaction between group and type of offers, Mann–Whitney U tests were performed with scores computing the difference of acceptance rate between each type of offers (i.e., unfair minus fair; unfair minus medium-fair; medium-fair minus fair). These analyses did not reach any significant results, all *p* > 0.05.

### Skin Conductance Response

**FIGURE 2:**
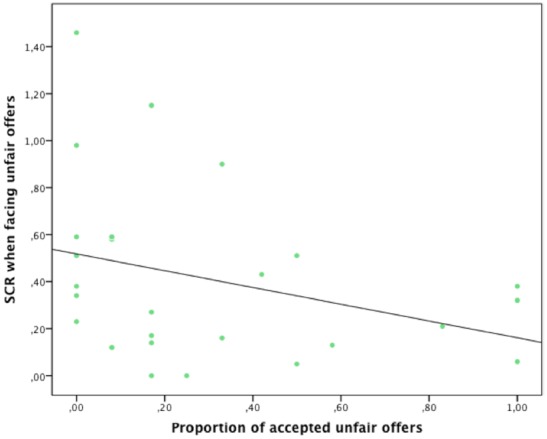
**In the alcohol-dependent group (*n* = 26), proportion of accepted unfair offers was negatively correlated with SCR amplitude triggered by unfair offers (Spearman Rho = –0.41, *p* = 0.04)**.

A Friedman test was performed separately in each group in order to examine the effect of the type of offers (fair vs. medium-fair vs. unfair) on SCR (in mS). These analyses showed no significant SCR differences, in each group (all *p* > 0.05), between the fair (control group: mean = 0.315, median = 0.254, 25th = 0.09, 75th = 0.42; alcohol group: mean = 0.41, median = 0.33, 25th = 0.138, 75th = 0.583), the medium-fair (control group: mean = 0.296, median = 0.18, 25th = 0.10, 75th = 0.47; alcohol group: mean = 0.288, median = 0.19, 25th = 0.008, 75th = 0.413) and the unfair offers (control group: mean = 0.292, median = 0.33, 25th = 0.071, 75th = 0.45; alcohol group: mean = 0.41, median = 0.33, 25th = 0.138, 75th = 0.583). We then performed correlation analyses (Spearman Rho) between SCR and proportion of acceptance, for each types of offer and each group separately. These analyses showed that, in the alcohol dependence group, proportion of acceptance is negatively correlated with SCR for the unfair offers (Spearman Rho = –0.41, *p* = 0.04; see Figure 2). This association did not reach significance in the control group. We observed no significant correlation for the fair and medium fair offers, in both control and AD groups.

Mann–Whitney U tests were then performed to examine between-groups differences on SCR according to the type of offers (fair, medium-fair, or unfair). These analyses showed no significant difference between AD and controls (all *p* > 0.05). Additional exploratory Mann–Whitney U tests were performed to examine between-groups differences on SCR according to decision types (accept or reject) for each type of offers (fair, medium-fair, unfair). These analyses revealed a significant difference between AD participants (*n* = 23; average rank = 21.75) and controls (*n* = 20; average rank = 14.44) for the rejection of the unfair offer (Mann–Whitney U statistic = 95.00, *Z* = –2.07, *p* = 0.038), indicating that AD (mean = 0.488, median = 0.445, 25th = 0.207, 75th = 0.589) exhibited a higher SCR amplitude before rejecting unfair offers, as compared to controls (mean = 0.28, median = 0.345, 25th = 0.075, 75th = 0.465). No other significant difference was observed.

## Discussion

In the present study, we examined the association between emotional state and unfairness sensitivity in recently abstinent AD individuals and in healthy controls while performing an UG. Emotional state was measured by autonomic reactivity as reflected by SCR.

At a behavioral level, AD rejected unfair offers more frequently than controls, which replicate findings from the previous UG study in alcohol-dependence (Brevers et al., 2013). At a psychophysiological level, the proportion of accepted unfair UG offers was negatively correlated with SCR, but in the AD group only. Hence, deciding to accept or reject an unfair offer during the UG was influenced by emotional psychophysiological reactivity in AD, but not in controls. In addition, AD exhibited higher SCR than controls for unfair offers, but only prior to rejecting the offer. Importantly, despite its statistical significance, the negative correlation between ADs’ proportion of accepted unfair UG offers and SCR remains moderate. In addition, between-groups analyses did not include participants who accepted all unfair offers (12 controls and three AD participants) and the number of observations (e.g., rejected vs. accepted offers) differed for each participants. Hence, current findings are preliminary and have to be interpreted with caution.

Present findings suggest that, in AD: the higher the SCR, the higher the rejection of unfair offers. Hence, because skin conductance reflects sympathetic tone (Bouscein, 1992; van’t Wout et al., 2006), the association between SCR changes and UG rejection rates might reflect increased affective-arousal activity when facing unfair UG offers in AD. In other words, present results suggest that unfair offers triggered an emotional state in ADs and, that as a consequence, they “punished” proposers by rejecting the offer, thus depriving the proposer of their greater share of the money (Nowak et al., 2000). By contrast, non-alcoholics were better able to act as rational economic agents (at both behavioral and psychophysiological levels) during the UG, which results in maximizing their monetary profits. However, in the present study, we used only one marker of emotional state (i.e., SCR). Hence, we cannot exclude that UG offers did not trigger significant emotional state in controls. In addition, present data offers no information on the valence associated with the SCR. Thus, we cannot infer that unfair offers triggered *negative* emotional state in AD. In this context, one option for future studies would be to make participants complete (after all trials of the UG) a subjective ratings of the emotional valence (e.g., from 1/negative to 9/positive) that they had experienced in each condition (i.e., fair, medium-fair, unfair) while playing the role of responder (e.g., Hewig et al., 2011).

Importantly, contradictory to our hypotheses and inconsistently with previous studies (van’t Wout et al., 2006; Hewig et al., 2011; but see Osumi and Ohira, 2009), SCR was not modulated by the degree of unfairness associated with UG offers. Moreover, the absence of a significant correlation between SCR and the proportion of accepted offers in control differs from previous studies which showed that the rejection of unfair UG offers is associated with greater SCR (Civai et al., 2010; Wu et al., 2013). One explanation for these discrepancies is that participants recruited for the present study where older (i.e., adults with age ranging from 20 to 51 years) than participants recruited in previous UG psychophysiological studies (i.e., young adults with mean age ranging from 20.10 to 23.56 years; van’t Wout et al., 2006; Osumi and Ohira, 2009; Civai et al., 2010; Hewig et al., 2011; Wu et al., 2013). Indeed, multiple lines of research have demonstrated that age is associated with lower negative affectivity (Pillutla and Murnighan, 1996). For instance, laboratory studies demonstrated that age is associated with lower negative experiential and physiological reactivity to laboratory emotion inductions (Levenson, 2000; Tsai et al., 2000; Labouvie-Vief et al., 2003). Hence, as compared to young adults, facing unfair offers might have less emotional impact on middle-aged adults, which could increase their level acceptance toward unequal offers. Another possible explanation for these findings is that, in the present study, no real money was awarded to the participants. As a result, the use of hypothetical money could have lowered participants’ emotional engagement during the UG task and decreased the validity of the observed SCR. Hence, one direction for future studies would be to examine the emotional correlates of unfairness sensitivity in alcohol dependence through the use of an experiential UG played with real money.

An implication of present findings is that AD might have difficult to cope with unfair situations triggered by (e.g.) social interactions. Nevertheless, additional studies are needed in order to examine whether—emotional and behavioral—reactivity to unfairness during the UG could predict relapse in abstinent AD. Indeed, sensitivity to frustration has been reported to impact alcohol consumption and relapse in abstinent AD patients (Muraven et al., 2002; Baars et al., 2013; Winward et al., 2014). Future studies should also examine psychophysiological responses to UG offers in different subtypes of AD. For instance, patients with high interoceptive awareness are at elevated risk of negative mood or stress-induced relapse (Wiens, 2005; Verdejo-Garcia et al., 2012). As a result, these individuals might exhibit increased emotional and behavioral reactivity to unfairness during the UG. Indeed, it has been evidenced that SCR responses is associated with UG offers’ rejection in people with high interoceptive abilities but not in individuals with poor interoceptive sensitivity (Dunn et al., 2012; see also Kirk et al., 2011). It would be also interesting to examine whether Cloninger Type 1—anxiety prone—alcoholism differ from Cloninger Type 2—impulsive—alcoholism at the UG. For instance, it has been highlighted that people differing in levels of anxiety showed distinct behavior patterns and autonomic neural responses during the UG (i.e., SCR to inequitable offers was correlated with levels of anxiety in individuals with high-trait anxiety, but not in the individuals with low-trait anxiety), and that impulsivity might be an additional moderating factors in UG offers acceptance (Wu et al., 2013). Brain-imaging studies could also shed light on specific processes underlying impaired social decision-making in alcohol-dependence. For instance, insular activation plays a key role in representing somatic states used to simulate the potential negative consequences of an action (Damasio, 1994; Craig, 2002, 2009), such as when people reject unfair UG offers at substantial cost to themselves (Sanfey et al., 2003; Grecucci et al., 2012; Harle et al., 2012). Hence, it would be interesting to examine if increased unfairness sensitivity in AD is underlined by increased insular activation during the UG.

In sum, AD exhibited a lower rate of acceptation for unfair offers during an UG task. Moreover, ADs’ tendency to accept or reject unfair UG offers was associated with the level of electrodermal response triggered by the offer. This association was not found in control participants. These findings offer a first evidence of hampered emotion regulation in alcohol dependence when facing a social context of unfairness. Nevertheless, these results remain preliminary and additional studies are needed in order to further examine the impact of emotion on unfairness sensitivity in AD.

### Conflict of Interest Statement

The authors declare that the research was conducted in the absence of any commercial or financial relationships that could be construed as a potential conflict of interest.

## References

[B1] American Psychiatric Association. (2013). Diagnostic and Statistical Manual of Mental Disorders, 5th Edn. Arlington, VA: American Psychiatric Publishing.

[B2] Baars M. Y. Müller M. J. Gallhofer B. Netter P. (2013). Relapse (number of detoxifications) in abstinent male alcohol-dependent patients as related to personality traits and types of tolerance to frustration. Neuropsychobiology 67, 241–248. 10.1159/000350483 23689792

[B3] Bechara A. (2005). Decision making, impulse control and loss of willpower to resist drugs: a neurocognitive perspective. Nat. Neurosci. 8, 1458–1463. 10.1038/nn1584 16251988

[B4] Beck A. T. Ward C. H. Mendelson M. Mock J. Erbaugh J. (1961). An inventory for measuring depression. Arch. Gen. Psychiatry 4, 561–571. 10.1001/archpsyc.1961.01710120031004 13688369

[B5] Bouscein W. (1992). Electrodermal Activity. New York, NY: Plenum.

[B6] Brevers D. Bechara A. Cleeremans A. Kornreich C. Verbanck P. Noël X. (2014). Impaired decision-making under risk in individuals with alcohol dependence. Alcohol. Clin. Exp. Res. 38, 1924–1931. 10.1111/acer.12447 24948198 PMC4115290

[B7] Brevers D. Noël X. Ermer E. Dabiri D. Verbanck P. Kornreich C. (2013). Unfairness sensitivity and social decision-making in individuals with alcohol dependence: a preliminary study. Drug Alcohol Depend. 133, 772–775. 10.1016/j.drugalcdep.2013.08.013 24021763

[B8] Camchong J. Endres M. Fein G. (2014). Decision making, risky behavior, and alcoholism. Handb. Clin. Neurol. 125, 227–236. 10.1016/B978-0-444-62619-6.00014-8 25307578

[B9] Camerer C. F. Thaler R. (1995). Anomalies: dictators, ultimatums, and manners. J. Econ. Perspect. 9, 209–219. 10.1257/jep.9.2.209

[B10] Canli T. Lesch K. P. (2007). Long story short: the serotonin transporter in emotion regulation and social cognition. Nat. Neurosci 10, 1103–1109. 10.1038/nn1964 17726476

[B11] Carmona-Perera M. Clark L. Young L. Perez-Garcia M. Verdejo-Garcia A. (2014). Impaired decoding of fear and disgust predicts utilitarian moral judgment in alcohol-dependent individuals. Alcohol. Clin. Exp. Res. 38, 179–185. 10.1111/acer.12245 24447115

[B12] Carmona-Perera M. Reyes del Paso G. A. Pérez-García M. Verdejo-García A. (2013). Heart rate correlates of utilitarian moral decision-making in alcoholism. Drug Alcohol Depend. 133, 413–419. 10.1016/j.drugalcdep.2013.06.023 23880247

[B13] Carmona-Perera M. Verdejo-García A. Young L. Molina-Fernández A. Pérez-García M. (2012). Moral decision-making in polysubstance dependent individuals. Drug Alcohol Depend. 126, 389–392. 10.1016/j.drugalcdep.2012.05.038 22749562

[B14] Civai C. Corradi-Dell’Acqua C. Gamer M. Rumiati R. I. (2010). Are irrational reactions to unfairness truly emotionally-driven? Dissociated behavioural and emotional responses in the ultimatum game task. Cognition 114, 89–95. 10.1016/j.cognition.2009.09.001 19786275

[B15] Craig A. D. (2002). How do you feel? Interoception: the sense of the physiological condition of the body. Nat. Rev. Neurosci. 3, 655–666. 10.1038/nrn894 12154366

[B16] Craig A. D. (2009). How do you feel—now? The anterior insula and human awareness. Nat. Rev. Neurosci. 10, 59–70. 10.1038/nrn2555 19096369

[B17] Damasio A. R. (1994). Descartes’ Error: Emotions, Reason, and the Human Brain. New York, NY: Avon Books.

[B18] Dunn B. D. Evans D. Makarova D. White J. Clark L. (2012). Gut feelings and the reaction to perceived inequity: the interplay between bodily responses, regulation and perception shapes the rejection of unfair offers on the ultimatum game. Cogn. Affect. Behav. Neurosci. 12, 419–429. 10.3758/s13415-012-0092-z 22618636 PMC3400033

[B19] Fehr E. Camerer C. F. (2007). Social neuroeconomics: the neural circuitry of social preferences. Trends Cogn. Sci. 11, 419–427. 10.1016/j.tics.2007.09.002 17913566

[B20] Fehr E. Gächter S. (2000). Cooperation and punishment in public goods experiments. Am. Econ. Rev. 90, 980–994. 10.1257/aer.90.4.980

[B21] Fehr E. Gächter S. (2002). Altruistic punishment in humans. Nature 415, 137–140. 10.1038/415137a 11805825

[B22] Feng C. Luo Y. J. Krueger F. (2014). Neural signatures of fairness-related normative decision making in the ultimate game: a coordinate-based meta-analysis. Hum. Brain Mapp. 36, 591–602. 10.1002/hbm.22649 25327760 PMC6869807

[B23] Grecucci A. Giorgetta C. Van’t Wout M. Bonini N. Sanfey A. G. (2012). Reappraising the ultimatum: an fMRI study of emotion regulation and decision making. Cereb. Cortex 23, 399–410. 10.1093/cercor/bhs028 22368088

[B24] Greene J. D. Sommerville R. B. Nystrom L. E. Darley J. M. Cohen J. D. (2001). An fMRI investigation of emotional engagement in moral judgment. Science 293, 2105–2108. 11557895 10.1126/science.1062872

[B25] Güth W. Schmittenberger R. Schwarze B. (1982). An experimental analysis of ultimatum bargaining. J. Econ. Behav. Organ. 3, 367–388. 10.1016/0167-2681(82)90011-7

[B26] Harle K. M. Chang L. J. van ‘t Wout M. Sanfey A. G. (2012). The neural mechanisms of affect infusion in social economic decision-making: a mediating role of the anterior insula. Neuroimage 61, 32–40. 10.1016/j.neuroimage.2012.02.027 22374480

[B27] Haselhuhn M. P. Mellers B. A. (2005). Emotions and cooperation in economic games. Brain Res. Cogn. Brain Res. 23, 24–33. 10.1016/j.cogbrainres.2005.01.005 15795131

[B28] Hewig J. Kretschmer N. Trippe R. H. Hecht H. Coles M. G. H. Holroyd C. B. (2011). Why humans deviate from rational choice. Psychophysiology 48, 507–514. 10.1111/j.1469-8986.2010.01081.x 20667034

[B29] Khemiri L. Guterstam J. Franck J. Jayaram-Lindsrtröm N. (2012). Alcohol dependence associated with increased utilitarian moral judgment: a case control study. PLoS ONE 7:e39882. 10.1371/journal.pone.0039882 22761922 PMC3386169

[B30] Kirk U. Downar J. Montague R. P. (2011). Interoception drives increased rational decision-making in meditators playing the ultimatum game. Front. Neurosci. 5:49. 10.3389/fnins.2011.00049 21559066 PMC3082218

[B31] Knafo A. Israel S. Darvasi A. Bachner-Melman R. Uzefovsky F. Cohen L. (2008). Individual differences in allocation of funds in the dictator game associated with length of the arginine vasopressin 1a receptor RS3 promoter region and correlation between RS3 length and hippocampal mRNA. Genes Brain Behav. 7, 266–275. 10.1111/j.1601-183X.2007.00341.x 17696996

[B32] Koenigs M. Tranel D. (2007). Irrational economic decision-making after ventromedial prefrontal damage: evidence from the ultimatum game. J. Neurosci. 27, 951–956. 10.1523/JNEUROSCI.4606-06.2007 17251437 PMC2490711

[B33] Kornreich C. Brevers D. Ermer E. Hanak C. Verbanck P. Campanella S. (2013). Polysubstance dependent patients display a more utilitarian profile in moral decision-making than alcohol-dependent patients, depressive patients and controls. Drug Alcohol Depend. 132, 434–440. 10.1016/j.drugalcdep.2013.03.005 23540448

[B34] Labouvie-Vief G. Lumley M. A. Jain E. Heinze H. (2003). Age and gender differences in cardiac reactivity and subjective emotion responses to emotional autobiographical memories. Emotion 3, 115–126. 10.1037/1528-3542.3.2.115 12899414

[B35] Levenson R. W. (2000). “Expressive, physiological, and subjective changes in emotion across adulthood,” in Psychology and the Aging Revolution: How We Adapt to Longer Life, eds Qualls S. H. Abeles N. (Washington, DC: American Psychological Association), 123–140.

[B36] Muraven M. l. Collins R. L. Nienhaus K. (2002). Self-control and alcohol restraint: an initial application of the self-control strength model. Psychol. Addict. Behav. 16, 113–120. 10.1037/0893-164X.16.2.113 12079249

[B37] Noël X. Bechara A. Brevers D. Verbanck P. Campanella S. (2010). Alcoholism and the loss of willpower: a neurocognitive perspective. J. Psychophysiol. 24, 240–248. 10.1027/0269-8803/a000037 21765575 PMC3136191

[B38] Noël X. Van der Linden M. d’Acremont M. Bechara A. Dan B. Hanak C. (2007). Alcohol cues increase cognitive impulsivity in individuals with alcoholism. Psychopharmacology 192, 291–298. 10.1007/s00213-006-0695-6 17279375

[B39] Nowak M. A. Page K. M. Sigmund K. (2000). Fairness versus reason in the ultimatum game. Science 289, 1773–1775. 10.1126/science.289.5485.1773 10976075

[B40] Osumi T. Ohira H. (2009). Cardiac responses predict decisions: an investigation of the relation between orienting response and decisions in the ultimatum game. Int. J. Psychophysiol. 74, 74–79. 10.1016/j.ijpsycho.2009.07.007 19646490

[B41] Pillutla M. M. Murnighan J. K. (1996). Unfairness, anger, and spite: emotional rejections of Ultimatum Offers. Organ. Behav. Hum. Decis. Process. 68, 208–224. 10.1006/obhd.1996.0100

[B42] Rilling J. K. Sanfey A. G. (2011). The neuroscience of social decision-making. Annu. Rev. Psychol. 62, 23–48. 10.1146/annurev.psych.121208.131647 20822437

[B43] Sanfey A. G. Rilling J. K. Aronson J. A. Nystrom L. E. Cohen J. D. (2003). The neural basis of economic decision-making in the ultimatum game. Science 300, 1755–1758. 10.1126/science.1082976 12805551

[B44] Saunders J. B. Aasland O. G. Babor T. F. de la Fuente J. R. Grant M. (1993). Development of the Alcohol Use Disorders Identification Test (AUDIT): WHO collaborative project on early detection of persons with harmful alcohol consumption-II. Addiction 88, 791–804. 10.1111/j.1360-0443.1993.tb02093.x 8329970

[B45] Spielberger C. (1983). Manual for the State-Trait Anxiety Inventory: STAI (Eorm I). Palo Alto, CA: Consulting Psychologists Press.

[B46] Trivers R. L. (1971). The evolution of reciprocal altruism. Q. Rev. Biol. 46, 35–57. 10.1086/406755

[B47] Tsai J. L. Levenson R. W. Carstensen L. L. (2000). Autonomic, subjective, and expressive responses to emotional films in older and younger Chinese Americans and European Americans. Psychol. Aging 15, 684–693. 10.1037/0882-7974.15.4.684 11144327

[B48] van’t Wout M. Kahn R. S. Sanfey A. G. Aleman A. (2006). Affective state and decision-making in the ultimatum game. Exp. Brain Res. 169, 564–568. 10.1007/s00221-006-0346-5 16489438

[B49] Verdejo-Garcia A. Clark L. Dunn B. D. (2012). The role of interoception in addiction: a critical review. Neurosci. Biobehav. Rev. 36, 1857–1869. 10.1016/j.neubiorev.2012.05.007 22659642

[B50] Wiens S. (2005). Interoception in emotional experience. Curr. Opin. Neurobiol. 18, 442–447. 10.1097/01.wco.0000168079.92106.99 16003122

[B51] Winward J. L. Bekman N. M. Hanson K. L. Lejuez C. W. Brown S. A. (2014). Changes in emotional reactivity and distress tolerance among heavy drinking adolescents during sustained abstinence. Alcohol. Clin. Exp. Res. 38, 1761–1769. 10.1111/acer.12415 24818520 PMC4047160

[B52] Wu T. Luo Y. Broster L. S. Gu R. Luo Y. J. (2013). The impact of anxiety on social decision-making: behavioral and electrodermal findings. Soc. Neurosci. 8, 11–21. 10.1080/17470919.2012.694372 22670854 PMC3632664

